# Non-aflatoxigenic *Aspergillus flavus* to prevent aflatoxin contamination in crops: advantages and limitations

**DOI:** 10.3389/fmicb.2014.00050

**Published:** 2014-02-10

**Authors:** Kenneth C. Ehrlich

**Affiliations:** Southern Regional Research Center, United States Department of Agriculture – Agricultural Research ServiceNew Orleans, LA, USA

**Keywords:** aflatoxin, *Aspergillus flavus*, biocontrol, food safety, recombination, maize, cottonseed, population diversity

## Abstract

*Aspergillus flavus* is a diverse assemblage of strains that include aflatoxin-producing and non-toxigenic strains with cosmopolitan distribution. The most promising strategy currently being used to reduce preharvest contamination of crops with aflatoxin is to introduce non-aflatoxin (biocontrol) *A. flavus* into the crop environment. Whether or not introduction of biocontrol strains into agricultural fields is enough to reduce aflatoxin contamination to levels required for acceptance of the contaminated food as fit for consumption is still unknown. There is no question that biocontrol strains are able to reduce the size of the populations of aflatoxin-producing strains but the available data suggests that at most only a four- to five-fold reduction in aflatoxin contamination is achieved. There are many challenges facing this strategy that are both short term and long term. First, the population biology of *A. flavus* is not well understood due in part to *A. flavus*’s diversity, its ability to form heterokaryotic reproductive forms, and its unknown ability to survive for prolonged periods after application. Second, biocontrol strains must be selected that are suitable for the environment, the type of crop, and the soil into which they will be introduced. Third, there is a need to guard against inadvertent introduction of *A. flavus* strains that could impose an additional burden on food safety and food quality, and fourth, with global warming and resultant changes in the soil nutrients and concomitant microbiome populations, the biocontrol strategy must be sufficiently flexible to adapt to such changes. Understanding genetic variation within strains of *A. flavus* is important for developing a robust biocontrol strategy and it is unlikely that a “one size fits all” strategy will work for preharvest aflatoxin reduction.

## DEVELOPMENT OF THE BIOCONTROL STRATEGY

Fungal growth on agricultural commodities, with or without mycotoxin production, does not occur in pure culture. Early studies found that aflatoxin production by *Aspergillus flavus* is reduced when it is cultivated with certain other fungi and bacteria (Ashworth et al., 1965; Weckbach and Marth, 1977; Wicklow et al., 1980, 1988; Horn and Wicklow, 1983; Ehrlich et al., 1985). The soil microbiome, mainly fungi and bacteria, affects the ability of the fungi to produce secondary metabolites (Ashworth et al., 1965). It has long been known that some plants are never contaminated with aflatoxin even though aflatoxin-producing species are present in the soil (Wogan, 1966). However, when tissues of these resistant plants are sterilized, *A. flavus* has no trouble producing aflatoxin on the tissue (Ehrlich and Ciegler, 1985). The ability of a fungus to compete for a host depends on many factors including pH, soil type, nitrogen and carbon availability, and water and mineral content (Eugenio et al., 1970). In 1975 an outbreak of *A. flavus* contamination of field corn in Iowa led to an unexpectedly low level of aflatoxin contamination of the crop (Ehrlich et al., 1985). Wicklow and others noticed that co-culture of *A. flavus* with *A. niger* caused a marked reduction in the formation of aflatoxin beyond a simple displacement of one fungus by the other (Wicklow et al., 1980). We found that co-cultivation of *A. flavus* with *P. oxalicum* had a similar effect on aflatoxin production but, in this case, not only were aflatoxin amounts reduced to a level not accountable by simple displacement, but a metabolite of *P. oxalicum*, secalonic acid, was also reduced (Ehrlich et al., 1985). We speculated that the co-cultivation of the two organisms caused a competition for ATP that is needed for one or more of the oxidation steps in secondary metabolite formation. The order of inoculation was also important for determining which of the two competing fungi was successful in reducing mycotoxin production. Inoculation with the *P. oxalicum* first greatly inhibited *A. flavus* production of aflatoxins even when eventual growth was similar. We also showed that *A. flavus* mutants reduced in their abilities to produce aflatoxins also showed similar competitor ability to that of *P. oxalicum* (Ehrlich, 1987). Recent work (see below) has offered a different interpretation of the mechanism behind these competition results (Sweany et al., 2011).

This early work supported the concept that competition with *A. flavus* isolates incapable of aflatoxin production could remediate aflatoxin contamination. This mode of biocontrol is currently the most widely used biocontrol method for reducing aflatoxin contamination of cereal crops in maize and cottonseed where aflatoxin contamination is a persistent problem for human and animal health (Wu and Khlangwiset, 2010a). Cotty and co-workers in the 1990s found that one particular non-aflatoxigenic *A. flavus* isolate (AF36) isolated from Arizona cottonseed, is an especially good competitor for reduction of aflatoxin content of cottonseed (Cotty and Bhatnagar, 1994). They determined that isolates from this VCG group were generally good as competitors against aflatoxin-producing isolates (Chang et al., 2012). We subsequently found that this isolate was unable to produce aflatoxin because of a point mutation in the polyketide synthase gene that is necessary for aflatoxin biosynthesis (Ehrlich and Cotty, 2004).

Cotty and co-workers developed a method to apply the non-aflatoxigenic strain to cotton-growing fields to prevent aflatoxin production by the wild-type aflatoxigenic populations present in the growing regions (Cotty, 2006; Cotty and Mellon, 2006). The method involves spreading non-aflatoxigenic *A. flavus* spores onto the field at particular times prior to harvest (Jaime-Garcia and Cotty, 2007). They assumed that addition of the non-aflatoxin producing strain would then allow it to out-compete the wild-type populations for access to the cottonseed and thereby displace the wild-type fungus. Therefore, based on this concept they called this strategy a “displacement” strategy for biocontrol of aflatoxin contamination (Jaime-Garcia and Cotty, 2009). Assuming this is true than the resulting treated field should never have to be treated again because, then, only the non-aflatoxigenic population of fungi would be present in the field. This method to prevent aflatoxin contamination is now in widespread use in Arizona for cotton fields and in other places in the southeast U.S for treatment of maize-growing areas. In other countries where aflatoxin contamination of maize is an endemic problem such as Kenya and parts of China, other strains have been discovered that are being used for aflatoxin remediation. In some cases the competing fungi are used as “cocktails” that include application of multiple strains of non-aflatoxigenic *A. flavus* (Wu et al., 2013).

There is a growing awareness that *A. flavus* also produces an indole tetramic acid mycotoxin, cyclopiazonic acid (CPA), under the same conditions that it produces aflatoxin (Chang and Ehrlich, 2011). CPA is a specific inhibitor of sarcoplasmic and endoplasmic reticulum calcium-dependent ATPase, an enzyme necessary for proper muscle contraction and relaxation. Therefore, non-aflatoxigenic competitor isolates incapable of production of this metabolite are being used instead of AF36 for reduction of aflatoxin levels in maize (Abbas et al., 2011). In a subsequent section we will discuss the possibility that other mycotoxins are produced by both the non-aflatoxigenic and aflatoxigenic populations that could contribute to mycotoxin contamination and cause toxic effects in humans and animals upon consumption.

Although the biocontrol strategy for aflatoxin remediation is increasingly being adopted world wide, there are several potential pitfalls that should be addressed These include the need to better understand the natural diversity of *A. flavus* populations in agricultural soil, the effects of climate change on both this diversity and on plant susceptibility, the ability of the introduced biocontrol strain to outcross with existing aflatoxin-producing *A. flavus*, the adaptation of certain *A. flavus* isolates for predominant growth on the plant rather than in the soil, the difficulty in timing the application or controlling the stability of the inoculum, how the introduction of the biocontrol strain affects the soil microenvironment, the potential damage to the plant from the introduced strain, and the need to better understand the entire *A. flavus* toxin burden that may result from *A. flavus* contamination beyond that of aflatoxin. In addition the cost of the biocontrol method and the potential need to continue reapplication seasonally must also be considered in weighing the benefits of biocontrol *A. flavus* as a means of reducing food and feed contamination with aflatoxin.

## EFFICACY OF BIOCONTROL BY NON-AFLATOXIGENIC ISOLATES OF *A. flavus*

More than 100 countries have enforced or proposed regulations for levels of aflatoxin in feeds and foods (Wu et al., 2013). Because these levels are so low, the regulations place a strong burden on grain intended for export. Some of these requirements are listed in **Table 1**. Normally maize contains only low levels of aflatoxin and usually meets these requirements (Yu et al., 2008), but in years with severe outbreaks of *A. flavus*, contamination levels can exceed 100–200 ppb (Johansson et al., 2006). In cottonseed grown in Arizona levels of aflatoxin frequently exceed the levels permitted for commerce and remediation by either diluting the contaminated meal with less contaminated grain or by chemical treatment to destroy aflatoxins is often necessary. **Table 2** presents a summary of results from several laboratories showing aflatoxin concentrations after treatments with several different biocontrol *A. flavus* and of several different crops. In some studies the reported reduction in aflatoxin content in treated versus untreated fields is as much as 20-fold (Dorner et al., 2003; Dorner, 2009, 2010; Abbas et al., 2011; Zanon et al., 2013). These data are for experimental laboratory studies where treatments were presumably done under optimized and highly controlled conditions. Generally a 5- to 20-fold reduction in aflatoxin levels would be sufficient for allowing the crop to meet the standards for consumption, but if the starting levels are particularly high, even a 20-fold reduction may not be enough. Recently, in several maize-growing regions in Kenya there have been reports of aflatoxin poisoning in humans (Probst et al., 2012; Wagacha et al., 2013; Yard et al., 2013). In these cases, ingestion was of maize that was contaminated after harvest by improper storage. It is not clear that a pre-harvest biocontrol strategy would be able to prevent such exposure and there may be simpler and more cost-effective methods to prevent ingestion of post-harvest contaminated maize.

**Table 1:** Allowable levels of aflatoxins in foods and feeds.

Country	Limit in PPB
France	0.1–10
Netherlands	0.02–5
Germany	5
Japan	10
Austria	0.2–1
United Kingdom	10
India	30
Malaysia	35
Mexico	20
United States	20

**Table 2:** Efficacy of biocontrol treatments.

Crop	Non-AF agent	Range of AF reduction % (treated control)	Reference
Maize	K49	83–98	Abbas etal. (2012)
	Afla-guard	9–75	Dorner (2009)
	Afla-guard	85–88	Dorner (2010)
Peanut	Afla-guard	89–96	Dorner etal. (2003)
	AFCHG2	75	Zanon etal. (2013)
Cotton	AF36	20–88	Cotty and Bhatnagar (1994)

## LIFE CYCLE AND DIVERSITY OF *A. flavus*

*Aspergillus flavus* is the most common species associated with aflatoxin contamination of agricultural crops (Cotty et al., 1994; Cotty, 1997). *A. flavus* is found in temperate and tropical regions in soil and, in agricultural areas, most commonly, on maize, cotton, tree, and ground nuts (Samuel et al., 2013) and less frequently on rice (Chen et al., 2013). *A. flavus* populations are highly diverse and their stability in the soil and on the plant is not well understood. An atoxigenic relative of *A. flavus*, *A.* *oryzae*, is widely used in soybean and rice fermentation (Chang and Ehrlich, 2010). It is now increasingly clear that *A.* *oryzae* is not a separate species but actually is only one of many examples of atoxigenic variants of *A. flavus *(Geiser et al., 2000; Chang et al., 2006). Other aflatoxin-producing fungi have been implicated in contamination of agricultural commodities. *A. parasiticus* has been associated with contaminations of peanuts in the United States (Horn, 2005), Argentina (Vaamonde et al., 2003), and West Africa (Ismail, 2001), but generally, the predominant contaminating organism is *A. flavus* (Cotty et al., 1994). *A. flavus *appears to be more invasive and out-competes *A. parasiticus *when both species are together in the soil. *A. nomius* is more rarely found in the soil, and usually is not associated with agricultural contamination episodes (Cotty et al., 1994; Bhatnagar et al., 2001; Cardwell and Cotty, 2002). Mis-identification of the contaminating organism, in some cases is possible. For example, in Thailand, some aflatoxin B-and G-producing organisms, found to be common in the soil resemble *A. flavus*, but have been conclusively identified as a new clade of *A. nomius *(Ehrlich et al., 2007a).

**FIGURE 1:**
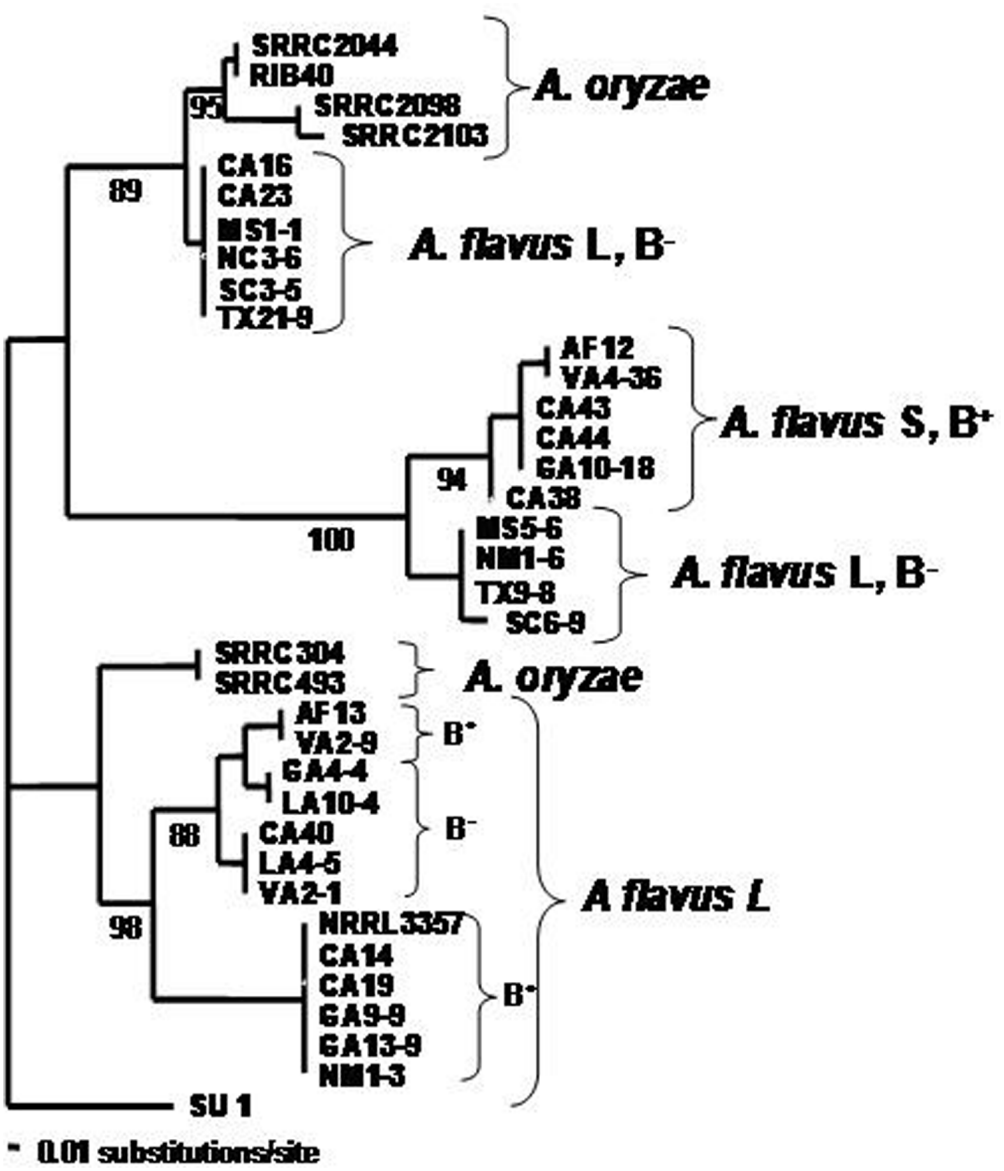
**Cladogram illustrating the diversity of *A. flavus* isolates selected from several different cotton and maize-growing areas in the United States.** RIB40 is the Japanese *A. oryzae* strain used for soy and rice fermentations.

*A. flavus* is a diverse assemblage of strains which include toxin-producing and non-toxigenic strains, sclerotial type variants, strains with variability in response to light, strains residing in multiple vegetative compatibility groups (VCGs), and strains with variable ability to colonize living plant tissue. A cladogram showing *A. flavus* diversity is shown in **Figure 1**. As a predominantly saprophytic fungus, *A. flavus* resides in the soil, but as an opportunist it is readily able to colonize most environments whenever there is a rich source of carbon and nitrogen. *A. flavus*’s diversity, therefore, appears to be an evolutionary response to its cosmopolitan distribution. Its main mode of replication is by asexual sporulation but under some conditions, *A. flavus* forms sclerotia, hardened masses of desiccated and melanized mycelia that are able to survive adverse environmental and nutritional conditions.

*Aspergillus* *flavus* soil populations contain isolates from two morphologically distinct sclerotial size variants, termed the L-strain [also called *A. flavus* Group IB (Geiser et al., 2000)] for isolates with average sclerotial size >400 μm and the S-strain (Group IA) for isolates with sclerotial size less that 400 μm (Cotty, 1997). Both S-and L-strains of *A. flavus* are found globally in maize-growing regions of the world. On typical laboratory growth media, when grown in the dark, S-strain isolates produce higher levels of aflatoxins, more abundant sclerotia, and fewer conidia. Atoxigenic S-strain isolates are very rarely found in natural environments (Orum et al., 1997). *A. flavus* lacks the ability to produce G-aflatoxins due to a gap in the cluster that includes a required cytochrome P450-encoding gene, *cypA*. The size of the deletion that causes loss of a portion of *cypA* is 1.5 kb for S-strain isolates and 0.8 kb for L-strain isolates. Differences in sclerotial morphology correlate with the differences between the S- and L-strain *A. flavus* in the size of the deletion in the *norB*-*cypA* gene (Ehrlich et al., 2004). Soil populations of *A. flavus* are typically composed of isolates from hundreds of different VCGs. Although frequent genetic exchange among these groups has not been observed, historical recombination in populations probably has occurred. Because the 0.8 kb**deletion in S-strain isolates is identical to the deletion in those *A. oryzae *isolates that possess most of the aflatoxin cluster, such isolates may have descended from a common ancestor that had the S-strain-type *norB*-*cypA* gene deletion (Chang et al., 2005). On average 30% of the *A. flavus* soil isolates in Arizona were identified as belonging to the S-strain (Cotty, 1997; Orum et al., 1997). Because S-strain isolates consistently produce more aflatoxin than L-strain isolates and aflatoxin production in this strain is not as strongly affected by nitrogen source, the concentration of S-strain isolates in the soil appears to be better correlated with major outbreaks of aflatoxin contamination in cotton-growing areas in Arizona and Texas (Orum et al., 1997; Jaime-Garcia and Cotty, 2006). Furthermore, up to 40% of the L-strain soil isolates of *A. flavus* found in Arizona and other regions of the United States (Horn and Dorner, 1999) were incapable of producing aflatoxins while S-strain isolates rarely were atoxigenic (Cotty et al., 1994). Interestingly, Two of the biocontrol strains used in the United States, AF36 and K49, have the S-strain type *norB-cypA *deletion which may correlate with their competitor abilities (Chang et al., 2012).

## POPULATION DYNAMICS OF *A. flavus* IN AGRICULTURAL ENVIRONMENTS

*Aspergillus flavus* was considered to be incapable of forming a sexual state and therefore was expected to maintain an entirely asexual life-style. Populations are divided into VCGs. Vegetative compatibility was believed to be a strong barrier to genetic exchange and in *A. flavus* was thought to be controlled by as many as 12 genetic loci (Bayman and Cotty, 1991; Ehrlich et al., 2007b). We found that *A. flavus* isolates from different VCGs formed genetically distinct groups suggesting that recombination is at most an infrequent event (Ehrlich et al., 2007b). Genetic isolation has been suggested by an additional study that found no evidence of gene flow between VCGs, including VCGs of opposite mating-type. Their results suggest that the VCGs diverged before domestication of agricultural hosts (> 10,000 year before the present; Grubisha and Cotty, 2009).

Recently *A. flavus* and many other presumed asexual fungi have been found to be capable of sexual reproduction, when grown in the dark under nutrient deprived conditions. *A. flavus*, as a heterothallic fungus, has two mating type loci, Mat1-1 and Mat1-2, maintained separately in homokaryotic isolates (Ramirez-Prado et al., 2008). Early evidence from genetic analysis suggested that *A. flavus* populations are able to undergo recombination (Geiser et al., 1998). Recent studies found that *A. flavus* in different VCG are able to outcross, and that VCG is not a strong barrier to sexual recombination (Olarte et al., 2012a). In fact such outcrossing among VCGs leads to new VCGs, and thereby, increased diversity (Olarte et al., 2012b). Most of these recombination studies have been done under laboratory conditions. Recombination can occur within conidia or sclerotia when they harbor multiple nuclei of different mating type. Fusion of nuclei containing different fluorescent markers revealed that, while conidial populations are predominantly homokaryotic, a small percentage can become heterokaryotic and, thereby, capable of recombination. The frequency of mating-type genes in the population was found to be correlated with recombination in the aflatoxin gene cluster.

Recombination has been detected between aflatoxigenic and non-aflatoxigenic *A. flavus* with some of the offspring regaining the ability to produce aflatoxins (Olarte et al., 2012a; Horn et al., 2013). Clearly, such recombination is a source of diversity within *A. flavus*. Because of this ability to recombine, it is critical to assess the frequency of such events in agricultural environments where atoxigenic biocontrol *A. flavus* have been introduced. A recent study found that, under an agricultural environment, a small percentage of the sclerotia that can form on contaminated maize can be heterokaryotic if the seed is contaminated with isolates of both mating types. Upon contact with non-sterile soil, these sclerotia can develop into ascocarps, the sexual reproductive developmental forms (Horn et al., 2013). A separate study found that soil populations in agricultural environments that were not treated with biocontrol *A. flavus* had approximately equal populations of fungi of both mating types. The population of fungi obtained from the plant (maize) was skewed to overrepresent isolates with Mat1-2 loci fungi (Sweany et al., 2011). These recent studies show that both asexual/sexual reproduction and ecological factors influence recombination.

It has been shown that the populations of *A. flavus* in an agricultural environment contain abundant amounts of non-aflatoxigenic *A. flavus *(Horn and Dorner, 1999). This suggests that loss of aflatoxin-producing ability in *A. flavus* could be a consequence of adaptation to a carbon-rich environment that makes the aflatoxin cluster less genetically stable. The ability to produce aflatoxins (and other mycotoxins) may give the fungi a long-term advantage over a non-aflatoxigenic biocontrol strain for survival in the soil, but in agricultural environments this adaptive pressure may be partially lost. Larger effective population sizes tend to increase mean population mutation and recombination rates (Hartl and Clark, 1997), further driving the evolution of new VCGs, some of which have lost aflatoxin-producing ability due to mutations within the biosynthetic cluster or due to large chromosomal deletions resulting in losses of entire telomeric regions (Chang et al., 2006). Since the aflatoxin and CPA clusters reside near the telomere of chromosome 3 in *A. flavus*, such mutations result in a high frequency of loss of aflatoxin and CPA-producing ability. The detection of linkage disequilibrium blocks in partial clusters indicates that recombination has played a large a role in cluster disassembly, and multilocus coalescent analyses of cluster and non-cluster regions indicate lineage-specific gene loss in *A. flavus *(Moore et al., 2007)*.*

The long-term fate of the non-aflatoxigenic biocontrol strain in the agricultural environment has not yet been fully addressed. A preliminary report found that in cotton fields treated with biocontrol *A. flavus* the introduced biocontrol isolate while the highly toxigenic strain S increased to reach an equilibrium in which the population of the biocontrol strain was about 10% that of the aflatoxin-producing isolate after 4 years. After only 1 year, the soil of treated fields had *A. flavus* populations with greater than 50% of the biocontrol isolate. This result suggests that long-term longevity of the biocontrol *A. flavus* could be an important consideration in establishing treatment protocols (Jaime-Garcia and Cotty, 2013). Survival of sclerotia in soil has been studied (Wicklow et al., 1993). After 36 months exposure to an agricultural soil, 68–100% of the sclerotia survived, with the main loss being due to nematode fungivory (Wicklow et al., 1993; McCormick, 2013). While soil sclerotia are largely stable in soil, the conidial inoculum is less stable and is subject to losses due to ingestion and degradation by soil bacteria and that formulations not using wheat or barley as a carrier are desirable (Accinelli et al., 2009).

## OTHER SECONDARY METABOLITE GENE CLUSTERS IN *A. flavus*

**FIGURE 2:**
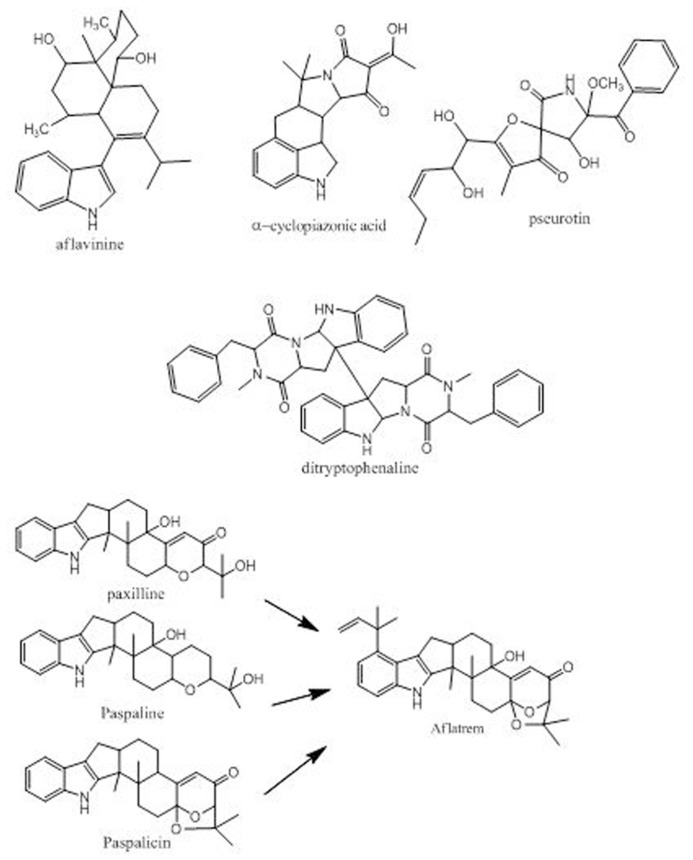
**Some known non-aflatoxin metabolites from *A. flavus***.

*Aspergillus flavus* is able to produce toxic secondary metabolites in addition to aflatoxins. This suggests that caution is needed in considering what isolates should be used as non-aflatoxigenic biocontrol agents (Rank et al., 2012). Among these secondary metabolites are the indole-diterpenes, aflatrem, paxillenes, paspalicines, and aflavinines, as well as is the ergot-like alkaloids CPA and pseurotin (**Figure 2**). While none of these metabolites is currently regulated as a food or feed contaminant, toxicity studies indicate that they could have neurotoxic and nephrotoxic effects on animals. Other metabolites are also frequent metabolites of *A. flavus*, including the Substance P neurotransmitter antagonist, ditryptophenaline. We have recently determined that some of these metabolites are produced in greater quantities in S strain *A. flavus* than in L strain and are produced by some of the non-aflatoxigenic competitor strains. The reported toxic effects on humans of ingestion of *A. flavus*-contaminated maize was growth retardation, immune suppression, and liver damage, the latter being manifested the most in people with hepatitis C infection (Probst et al., 2007; Probst et al., 2010; Wu and Khlangwiset, 2010b). These toxic effects have usually been ascribed to ingestion of aflatoxins. We suggest that simultaneous ingestion of other toxic *A. flavus* metabolites may contribute to these observed toxicities in people who have eaten aflatoxin-contaminated maize. The S morphotype *A. flavus*, the *Aspergillus* strain is most associated with the recent outbreaks of toxicity to humans in Kenya and Nigeria and may be far more toxic than the L strain (Donner et al., 2009; Mehl and Cotty, 2010; Probst et al., 2010). *A. flavus* is also able to produce metabolites that are usually not considered to be particularly toxic but could affect animal health. These include metabolites such as orcellinic acid, aspergillic acid and kojic acid (Varga et al., 2012) as well as iron-chelating siderophores similar to ferricrocin (Wallner et al., 2009). What effect these additional metabolites might have on animal and plant health is unknown.

## GLOBAL WARMING AND BIOCONTROL

Global warming has increased daily high temperatures in the mid west and northern maize-growing regions of the United States and Canada. The resulting temperatures are predicted to eventually resemble those in the southern United States where aflatoxin contamination of maize is a frequent problem. Aflatoxin contamination of mid west maize has not been recognized as a problem as yet. Besides temperature shifts, global warming can cause climate changes which result in more unpredictable weather problems for agricultural areas. Aflatoxin contamination events are more prevalent during times of high heat and drought, which may stress the host plant thereby facilitating *A. flavus* infection (Schmidt-Heydt et al., 2009; Roze et al., 2012; Mohale et al., 2013; Reverberi et al., 2013). Fungal stress has been correlated with increased expression of genes involved in both secondary metabolism production and sexual recombination as discussed above. Agricultural areas experiencing drought often suffer aflatoxin contamination outbreaks, and unpredictable changes in climate that result in drought may occur with increased frequency. Currently, incidences of aflatoxin contamination of crops are limited to tropical and sub-tropical areas (between latitudes 40°N and 40°S) around the world (Samuel et al., 2013). Because the average global surface temperature has increased by 0.8°C since 1901, with most of that increase occurring in the last 30 years, it is possible that by the end of the 21st century the favorable climate for aflatoxin contamination may encompass more of the maize-growing regions of the U.S. and outbreaks will become more frequent in occurrence.

Another potential consequence of climate change is that the biocontrol strain could be an inadvertent cause of increased damage to the plant, especially if growing conditions are less favorable for cultivation. Concomitantly, changes in the soil environment and its microbiome due to temperature elevation, could also subject the crop to increased damage. Understanding genetic variation within strains of *A. flavus* is important for developing a robust biocontrol strategy and it is unlikely that a “one size fits all” strategy will work.

## CONCLUSION

The ultimate goal for using non-aflatoxigenic *A. flavus* as a biocontrol agent should be the long-term protection of crops against aflatoxin contamination. Current strategies utilize a program of annual re-application of biocontrol strains, and the fate of the biocontrol strains after one growing season is still unknown. Even a low rate of recombination for aflatoxigenic fungi could be significant for future food safety. There exist other challenges to the biocontrol strategy for remediation of aflatoxin contamination. The inherent diversity of *A. flavus* populations makes a biocontrol strategy more difficult because *A. flavus* populations differ in their abilities to produce aflatoxins and other toxic secondary metabolites. Some of these other secondary metabolites could be important for assessing the full toxic burden when grains contaminated with *A. flavus *are ingested*. *Climate change could increase stress on the plant and the fungus and environmental stress could increase plant susceptibility to the fungus and is a known inducer of secondary metabolite production. Stress could also affect the ability of the fungus to outcross with native populations of *A. flavus*. Also, in use of the biocontrol *A. flavus* care must be taken to prevent undue crop damage or damage to the soil microflora that might result.

## Conflict of Interest Statement

The author declares that the research was conducted in the absence of any commercial or financial relationships that could be construed as a potential conflict of interest.
